# Bradykinin and the Pathogenesis of Hereditary Angioedema

**DOI:** 10.1097/WOX.0b013e318216b7b2

**Published:** 2011-04-15

**Authors:** Allen P Kaplan

**Affiliations:** 1 Medical University of South Carolina, Charleston, South Carolina

## 

Hereditary angioedema (HAE) is a term coined for a familial form of potentially life-threatening angioedema first described by William Osler in 1888. It is inherited as an autosomal dominant and the older literature often employs the term angioneurotic edema because it was believed that swelling could be a consequence of an emotional disorder. This type of angioedema, in particular, often included family histories of death by asphyxiation so that family members appeared "neurotic," that is, overly fearful, anxious, depressed, and so on. It also turns out that episodes of angioedema may well be triggered by emotional events in addition to other known triggers such as trauma, infection, and estrogen-containing hormones, but the biochemical explanation for this is not yet known. However, the conception of the disorder changed in 1953 when Donaldson and Evans discovered that it is due to a mutation in a plasma enzyme inhibitor known as C1 inhibitor (C1 INH) [1]. It was then assumed that some vasoactive mediator from the complement system was the proximate cause of the angioedema.

The next era dealt with attempts to identify this vasoactive factor. It then became clear that his inhibitor could inactivate enzymes that are not part of the complement cascade. The first discovery was that it inhibited plasma kallikrein [2], an enzyme that cleaves a plasma kininogen to produce bradykinin. At that point, no one seriously considered bradykinin as a possible cause of the swelling. The focus was on a kinin thought to be derived from the complement cascade termed C2 kinin. The key experiment, published in a nonrefereed journal [3], was that activated C1 will cleave C2 (in the presence of C4 as a cofactor) to yield C2a + C2b, and that plasmin digestion of C2b would produce a kinin that could be distinguished from bradykinin by its inactivation by tryptic digestion. It could be assayed by contraction of an estrous rat uterus or with lesser sensitivity (but greater reliability), employing a guinea pig ileum. This observation was never confirmed by other workers but additional observations seemed to substantiate the idea. Studies of vascular permeability in human and rabbit skin indicated that injection of activated C1 led to increased vascular permeability that required C2; the swelling was augmented when skin testing of patients with hereditary angioedema was compared with normal controls [4]. There was even one publication by Donaldson [5] suggesting a linkage between activated Factor XII (activated Hageman factor) and activation of the complement cascade so that simultaneous production of bradykinin (inhibited by Factor XII activation) and the C2-kinin seemed possible.

During the years 1972-1990 I was studying the mechanism of activation of the plasma bradykinin cascade. Two forms of activated Factor XII were described, that is, Factor XIIa at about 80 kd and Factor XII fragment (XIIf) at 28.5-30 kd [6,7]; soon thereafter, the Factor XII native protein was purified [8]. Factor XIIa or Factor XIIf was shown to be required for kallikrein activity to be produced in plasma; prekallikrein was purified [9], and its mechanism of conversion to kallikrein described. A controversy regarding the number of kininogens in plasma was resolved to indicate that there is a high molecular weight kininogen (HK) that is rather selectively cleaved by plasma kallikrein [10,11] to yield bradykinin, whereas low molecular weight kininogen is preferentially cleaved by tissue kallikrein to produce lysylbradykinin (kallidin). About 80% of plasma kallikrein was found to circulate as a complex with HK [12] and the distal light chain segment of HK was shown to be the feature that distinguishes it from low molecular weight kininogen (LK). This HK domain has the site of prekallikrein binding [13,14] and the site responsible for the cofactor activity of HK, which accelerates the conversion of prekallikrein to kallikrein and also conversion of coagulation factor XI to factor XIa [14]. Activation of the bradykinin-forming cascade was known to occur upon binding to negatively charged surfaces (eg, a glass test tube to clot blood) and this acceleration by surface binding was studied in some detail [15,16]. Factor XII was shown to autoactivate upon binding [17] so a molecular mechanism for initiation of the cascade seemed to be in place. From the onset of these studies, in the late 1970s, I was of the opinion that bradykinin is the mediator of the swelling seen in hereditary angioedema and that C2 kinin might be an artifact. My bias was based on a conversation with Frank Austen that occurred when the C2 kinin data were presented at a plenary session of The Association of American Physicians and I was seated in the audience. A rat uterus-based bioassay was used to demonstrate the new kinin and I had been trying to use such an assay to measure bradykinin for months. Its baseline was poor, it contracted when agonist (any agonist, at times including the buffer control) was added to the bath solution, and I could not be sure what was real and what was not. I switched to using a guinea pig ileum. I looked at the data being presented, I turned to Frank Austen, who was sitting next to me, and exclaimed, "That's an artifact!" I could not, however, explain the C2-dependent increase in vascular permeability reported by skin testing and am not sure I could do so even now. One issue is that anything injected into hereditary angioedema skin will produce bradykinin because of the trauma. It was by then clear that all enzymes required for the production of bradykinin are inhibited by C1 inhibitor [18] and that active kallikrein was demonstrable within induced blisters of HAE patients [19].

Nevertheless, in 1983 I decided to try to reproduce these experiments demonstrating plasmin cleavage of C2a or C2b to generate a kinin-like molecule. We activated C1 by immune complexes in the presence of C4 and C2, added plasmin, and could not demonstrate any kinin [20]. By contrast, incubation of HAE plasma, collected in EDTA to inhibit kininases, generated increasing amounts of bradykinin even in the absence of an initiating surface [20], whereas normal plasma generated nothing. Thus, plasma deficient in C1 INH was unstable and activated seemingly spontaneously to generate bradykinin upon in vitro incubation. We concluded that C2-kinin is not present, implied that it does not exist, and stated that bradykinin is the likely mediator of swelling in HAE. A subsequent publication found a 25 amino acid peptide with permeability increasing properties within the C2 amino acid sequence, but it is not released by any known peptide [21].

Because C2-kinin and bradykinin were the only 2 possibilities considered as possible mediators of the swelling of HAE and the data upon which C2-kinin was based could not be reproduced, bradykinin would be the "answer." But not being mathematicians (who accept disproving 1 of 2 possible choices as answering the question), this conclusion was not accepted until an additional 15 years passed, even after the nonexistence of C2-kinin was confirmed by a second study [22]. Finally, years later bradykinin was accepted to be the cause of the swelling of HAE after the following studies were reported [1]. A confirmation of our 1983 paper demonstrated that bradykinin is the only vasoactive kinin generated in HAE plasma assayed by vascular permeability or radioimmunoassay and anti-C2 could not prevent the phenomenon [23,2]. A rodent model in which C1 INH was knocked could be "cured" not only by repleting the C1 INH but also by knocking out the bradykinin B-2 receptor [24,3]. Bradykinin levels are elevated locally at the site of swelling in HAE [25,4]. A unique family in which a mutation of C1 INH renders it inactive on activated C1 but normally active as an inhibitor of the enzymes of the bradykinin-forming cascade (ie, Factor XIIa, Factor XIIf, and kallikrein) has no episodes of angioedema in any family members [26]. Curiously, review articles during this period often mentioned C2-kinin as a viable alternative to bradykinin as the key mediator, and I wrote an editorial entitled "Does C2-kinin exist," indicating, in detail, why it does not [27]. Until someone else shows it existence, and that it has some role in HAE based on experimental evidence, it should not be mentioned in anything but an historical context and it should certainly not be included in diagrams dealing with the pathogenesis of HAE.

The notion that bradykinin is the mediator of HAE has lead to therapeutic advances that specifically target the Factor XII-prekallikrein HK-bradykinin cascade. Of course, repleting C1 INH by infusion can be employed to control acute episodes of swelling and for prophylaxis [28]. When prophylaxis is considered, C1 INH infusion may replace attenuated androgens primarily because it eliminates the possibility of cumulative androgen side effects, although it is far more costly. C1 INH does require intravenous infusion but having home access to treatment is an important step forward. Perhaps a subcutaneous preparation will be seen in the future as has occurred with intravenous immunoglobulin. Therapy for acute episodes of swelling now include ecallantide, a plasma kallikrein inhibitor [29], and Icatibant [30], a B-2 bradykinin receptor antagonist. These have a short half-life and therefore cannot be used for prophylaxis. They are administered subcutaneously and can be repeated within an hour should control of symptoms be suboptimal. Both are quite effective, particularly if given early at the onset of an episode, and can abort symptoms of peripheral angioedema, gastrointestinal attacks, and laryngeal edema. At present, ecallantide is approved in the United States and Icatibant is approved in Europe. The efficacy of these drugs add to the cumulative data demonstrating this bradykinin-forming cascade as the one responsible for symptoms despite the fact that the complement is activated. In fact, C1 is unstable in the absence of C1 INH so that C4 is continually being cleaved, and if synthesis does not keep up, a low C4 level will result. Thus, quantitation of C4 is a good screening determination, along with measurement of C1 INH by protein and function C4 is low 95% of the time in HAE patients who are asymptomatic; C2 remains normal. However, during an attack of swelling formation of Factor XIIf [31] directly activates the C1r subcomponent of complement so that further lowering of C4 occurs during episodes of swelling which approaches zero, and C2 levels decrease. This is the main connection between the bradykinin-forming cascade and complement activation.

New observations requiring more work in terms of their relationship to HAE include the following [1]: Prekallikrein is an enzyme (ie, it stoichiometrically digests the HK to which it is bound without being converted to kallikrein) and this reaction is inhibited by C1 INH [32,2]. Heat shock protein 90 derived from endothelial cells will interact with the prekallikrein-HK complex [33] and stoichiometrically convert prekallikrein to kallikrein. Thus, initiation of bradykinin formation and attacks of swelling might result from endothelial cell activation and HSP 90-dependent conversion of prekallikrein to kallikrein and not begin with Factor XII activation. However, the kallikrein that forms will then activate Factor XII secondarily so that the normal cascade for bradykinin generation can proceed. Because complexes of gC1qR-cytokeratin 1 and cytokeratin-u-PAR at the cell surface bind Factor XII and HK (to which prekallikrein is bound) at the cell surface [34], activation of Factor XII upon binding to gC1qR (acting as a cell "surface") can initiate bradykinin formation in the standard fashion [35,36] so that initiation might involve alterations in the gC1qR-Factor XII interaction. Further work along these lines may help elucidate many of the unknown aspects of HAE--What starts attacks? Why are they localized to certain areas? Does activation really begin with Factor XII or does some kallikrein form first? Can emotional stress really trigger an episode? If so, how?
